# Lipoprotein(a): the perpetual supporting actor

**DOI:** 10.1093/eurheartj/ehy385

**Published:** 2018-07-14

**Authors:** Baris Gencer, François Mach

**Affiliations:** Cardiology Division, Department of Specialties in Medicine, Geneva University Hospitals, Rue Gabrielle-Perret Gentil 4, 1211 Geneva 14, Switzerland

## Abstract

**Figure:**
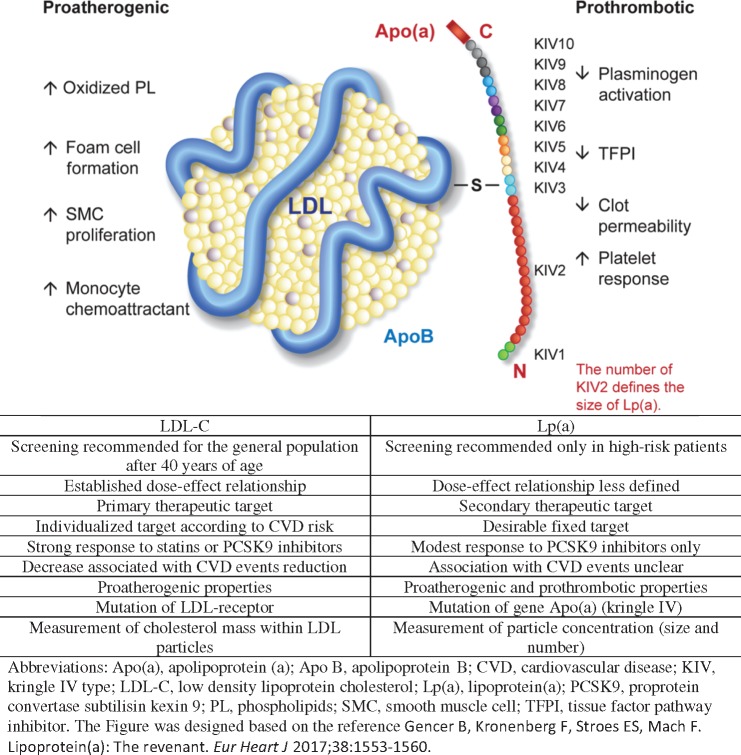

**This editorial refers to ‘Cardiovascular disease risk associated with elevated lipoprotein(a) attenuates at low low-density lipoprotein cholesterol levels in a primary prevention setting’^†^, by R. Verbeek *et al.*, on page 2589.**

In this issue of the *European Heart Journal*, Verbeek *et al.* investigated the risk pattern of Lipoprotein(a) [Lp(a)] across a wide range of values among two large community cohorts, as well as potential interactions with LDL-C levels.^1^ Data were derived from the well-known Copenhagen City Heart Study (CCHS), consisting of 9448 individuals, and the European Prospective Investigation of Cancer (EPIC)-Norfolk study, consisting of 16 654 individuals, in the primary prevention setting with available measurements for Lp(a) and LDL-C. The clinical primary outcome of cardiovascular disease (CVD) events was defined as coronary heart disease death, non-fatal myocardial infarction, and fatal or non-fatal stroke. Authors first categorized Lp(a) values at baseline into high (≥ 80th percentile) and normal (<80th percentile) groups using 50 mg/dL as a threshold, as recommended by the guidelines for clinical implications.^2^ In the EPIC-Norfolk cohort, patients’ CVD risk was lowest when associated with Lp(a) values <80th percentile and LDL-C <2.5 mmol/L, while patients’ CVD risk increased with higher LDL-C levels, reaching hazard ratios (HRs) of 1.61 [95% confidence interval (CI) 1.29–2.00, *P* < 0.001] for LDL-C ≥ 5.5 mmol/L. For patients with Lp(a) values ≥ 80th percentile, the risk for CVD events followed the same pattern, with an HR of 2.17 (95% CI 1.58–2.98, *P* < 0.001) for LDL-C values ≥ 5.5 mmol/L. These results corroborated with those of the CCHS study: HR for CVD was 1.42 (95% CI 1.15–1.74) for patients with Lp(a) values < 80^th^ percentile and LDL-C ≥ 5.5 mmol/L, and increased to HR 2.34 (95% CI 1.65–3.35, *P* < 0.001) for patients with Lp(a) values ≥ 80th percentile and LDL-C ≥ 5.5 mmol/L. HR for CVD risk demonstrated the highest level of significance (*P* < 0.001) for patients with Lp(a) values ≥ 80th percentile and LDL-C ≥ 5.5 mmol/L, compared with patients with Lp(a) values < 80th percentile and LDL-C < 2.5 mmol/L. Risk estimates followed the same pattern when using the threshold of 50 mg/dL: for patients with Lp(a) ≥ 50 mg/dL and LDL-C < 2.5 mmol/L, HR was 2.56 (95% 1.94–3.39) and 1.71 (95% CI 1.32–2.22) in the EPIC-Norfolk and the CCHS cohort, respectively, when compared with subjects with Lp(a) < 50 mg/dL and LDL-C < 2.5 mmol/L. Of note, for a same given level of LDL-C, CVD risk increased by 40–50% when Lp(a) values were high (≥ 50 mg/dL or ≥ 80th percentile).

Verbeek *et al.*’s article further highlights that high Lp(a) levels are also closely associated with adverse clinical outcomes in subjects with low LDL-C values. For instance, HR was 1.44 (95% CI 1.16–1.78, *P* < 0.01) in the group with LDL-C values ranging between 2.5–3.49 mmol/L in the EPIC-Norfolk cohort. On the other hand, in the relatively small group of patients with LDL-C < 2.5 mmol/L (< 10% of the cohort sample size) and high Lp(a), the association of CVD risk with Lp(a) levels was strongly attenuated: HR 1.11 (95% CI 0.77–1.59) and 1.08 (95% CI 0.85–1.38) in the EPIC-Norfolk and the CCHS cohort, respectively. However, the rate of CVD events did increase by more than 100% when high levels of Lp(a) were associated with higher LDL-C values (≥ 5.5 mmol/L). The findings of this large observational study add new evidence to the role of Lp(a) as a potential independent CVD risk factor, and suggest that the risk is highest when both Lp(a) and LDL-C values are elevated. HRs are, on the other hand, modest when taken individually for Lp(a). The clinical implications of these findings for medical practice remain still to be assessed, and it is also unclear whether Lp(a) can be added as an incremental value for CVD risk prediction beyond traditional risk factors.

Previous studies, including a large meta-analysis, have suggested that the risk of CVD increased according to Lp(a) concentration, after adjustment for sex and age.^3^^,^^4^ In addition, Mendelian randomization data have shown that genetic mutations for Lp(a) increase the risk of CVD, reinforcing the likely causal association between the two.^5^ A prospective cohort study including data from 46 200 individuals from the Copenhagen General Population Study showed that for patients with familial hypercholesterolemia (FH), the highest risk of CVD was associated with Lp(a) values ≥ 50 mg/dL (HR 5.3, 95% CI 3.6–7.6), and CVD risk remained high with normal Lp(a) values (HR 3.2, 95% CI 2.5–4.1) when compared with the reference group of subjects without FH and Lp(a) values ≤ 50 mg/dL.^6^ In the GENdEr and Sex determinantS of cardiovascular disease: from bench to beyond-Premature Acute Coronary Syndrome (GENESIS-PRAXY) study, including 939 individuals with premature acute coronary syndromes, patients with high Lp(a) were more likely to have high LDL-C values, with a significant synergistic interaction and an increasingly strong association for LDL-C thresholds > 3.5 and 4.5 mmol/L.^7^ The strength of Verbeek *et al.*’s study is that it expressly assessed CVD risk associated with Lp(a) levels according to different LDL-C cut-offs, with adequately powered sample sizes across all strata of the cohorts.

The results reported in Verbeek *et al.*’s study are valid for the primary prevention setting. Evidence is more controversial in secondary prevention and for patients on statins. In the Rosuvastatin Versus Atorvastatin (SATURN) trial, baseline and follow-up Lp(a) levels were not associated with changes in per cent atheroma volume as measured with ultrasound.^8^ LDL-C decreased from 114 mg/dL to 60 mg/dL, while Lp(a) remained unchanged (17.4 mg/dL and 16.5 mg/dL, respectively) with statin therapy. The cut-off of 50 mg/dL for Lp(a) was neither protective nor a risk factor for disease progression.^8^ These data suggest that Lp(a) is not a good marker for the estimation of residual CV risk after initiation of statin therapy. A second subanalysis of the dal-Outcomes trial, including 4139 acute coronary syndrome patients treated with statins, did not show any association between Lp(a) concentrations and major adverse outcomes: for a doubling of the dose of Lp(a), the level of risk for CVD was stagnant (HR 1.01, 95% CI 0.96–1.06, *P*=0.66).^9^ Similar results were also reported in three different pooled studies with 6708 subjects known for coronary artery disease: odds ratios (ORs) were 1.03 (95% CI 0.96–1.11) for each increase in log-transformed SD of Lp(a) or by quintile (highest vs. lowest OR 1.05, 95% CI 0.83–1.34).^10^

## Function of Lp(a)

What is the difference between Lp(a) and LDL-C? This key question is addressed in *Take home figure*. Lp(a) particles have two major and distinct components: (i) a structure similar to an LDL particle containing Apolipoprotein (Apo) B and (ii) a glycoprotein [Apo(a)] that is similar to plasminogen.^3^^,^^11^ The gene variations of Lp(a) mainly determine the size of Apo(a) via the number of kringle IV repetitions and not the LDL part. Besides a pro-atherogenic effect similar to that of LDL-C, which generates inflammation or cholesterol deposition in vessel walls,^12^ Lp(a) has additional pro-thrombotic properties that could potentially explain the associated increase in CVD risk for the same level of LDL-C in primary prevention.^3^ It is unclear at which point antithrombotic treatments could attenuate this phenomenon. For this reason, and as previously mentioned, Lp(a) is not a good surrogate marker in secondary prevention, or for patients treated with statin or antithrombotic therapy.

**Take home figure:**
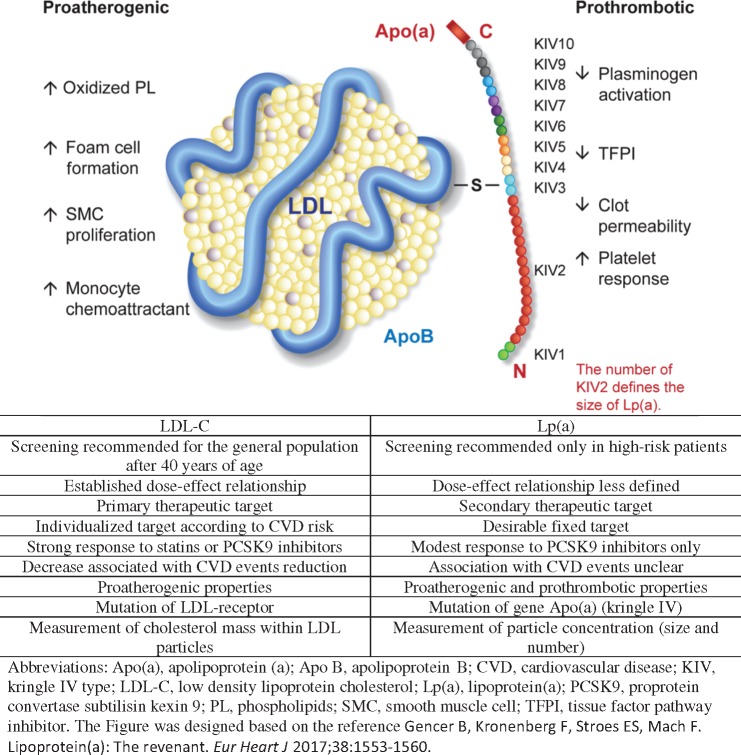
Comparison between LDL-C and lipoprotein(a).

## Lp(a): what do guidelines tell us?

The 2010 consensus document on Lp(a) from the European Society of Cardiology proposed a linear association between Lp(a) levels and CVD events,^13^ which might be too simplistic given the level of interaction and modification that LDL-C levels appear to have on cardiovascular risk. Measurement of Lp(a) is recommended in clinical practice in the following settings: (i) premature CVD, (ii) FH, (iii) a family history of CVD or elevated Lp(a), and (iv) recurrent CVD despite optimal statin therapy and ≥ 5% 10 year risk of fatal CVD according to Systematic COronary Risk Evaluation (SCORE).^2^ The treatment goal for CVD patients is first to lower LDL-C levels and reach desirable Lp(a) levels < 50 mg/dL. However, to date, there is no evidence that lowering Lp(a) really does improve clinical outcomes or that this molecule is a critical target to decrease residual risk. Statin treatment is not associated with a reduction of Lp(a), as opposed to PCSK9 inhibitors, which have been demonstrated to significantly reduce Lp(a) by 20–30%. In the case of proprotein convertase subtilisin kexin 9 (PCSK9) inhibitors, Lp(a) reduction is systematically correlated with LDL-C reduction, and it is most probably the increased levels of LDL-receptor expression typically induced by PCSK9 inhibitors that lead to subsequent lowering of circulating Lp(a) levels.^14^ The beneficial effect of PCSK9 inhibition on clinical events therefore seems to be mediated by reductions in LDL-C levels, while it remains unclear what benefit the concomitant decrease of Lp(a) might have.^15^ Recently, treatment with antisense oligonucleotides targeting Apo(a) in persons with elevated Lp(a) has shown very impressive results in lowering Lp(a) levels (≥ 70–80% decrease).^16^^,^^17^ A clinical study of cardiovascular outcomes is now required to evaluate the impact of specifically reducing Lp(a). A phase 2B trial will start to recruit patients with elevated Lp(a), defined as Lp(a) ≥ 60 mg/dL, and established CVD to test the efficacy and safety of ISIS 681257 administered subcutaneously with a target sample size of 270 participants (ClinicalTrials.gov identifier NCT03070782).

In conclusion, for more than three decades now, Lp(a) has been explored in various mechanistic and clinical studies, but continues to take the role of perpetual supporting actor as a secondary or exploratory target, since no therapy has yet succeeded in specifically lowering Lp(a) without at the same time lowering LDL-C. Recent developments might finally change the scenario by highlighting Lp(a)’s independent role in cardiovascular risk reduction. Lp(a) might yet receive a leading actor Oscar nomination after all.

## Footnotes

^†^ doi:10.1093/eurheartj/ehy334.
